# Development and Validation of a Stability-Indicating RP-UPLC Method for Determination of Rosuvastatin and Related Substances in Pharmaceutical Dosage Form

**DOI:** 10.3797/scipharm.1201-09

**Published:** 2012-03-26

**Authors:** Harshal Kanubhai Trivedi, Mukesh C. Patel

**Affiliations:** 1 Analytical Research Lab, Cadila Pharmaceutical Ltd., Dholka-387 810, Gujarat, India; 2 P.S. Science and H.D. Patel Arts College, S.V. Campus, Kadi-382 715, Gujarat, India

**Keywords:** Rosuvastatin calcium, Method validation, Forced degradation, Hyperlipidemia, Impurities, Liquid Chromatography, Rosuvastatin tablets, UPLC

## Abstract

A stability-indicating reversed phase ultra performance liquid chromatographic (RP-UPLC) method was developed for the determination of related substances in rosuvastatin calcium (ROSV) tablet dosage form. The chromatographic separation was achieved on an Acquity BEH C18 (100 mm × 2.1 mm, 1.7 μm) column with mobile phase containing a gradient mixture of solvent-A (0.1% trifluoroacetic acid) and solvent-B (methanol). The eluted compounds were monitored at 240 nm and the run time was 10.0 min. Degradation behavior of the ROSV was studied under various degradation stress conditions. Four major unknown degradation products (late eluting impurities) were found in acid stress condition and two unknown degradation products were found in oxidative stress condition. The developed method separates (six) unknown impurities, (three) known impurities and ROSV substance from each other, providing the stability-indicating power of the method. The developed RP-UPLC method was validated according to the International Conference on Harmonization (ICH) guidelines. The developed and validated RP-UPLC method is LC-MS compatible and can be applied for identification of eluted unknown impurities of ROSV.

## Introduction

**Fig. 1.**
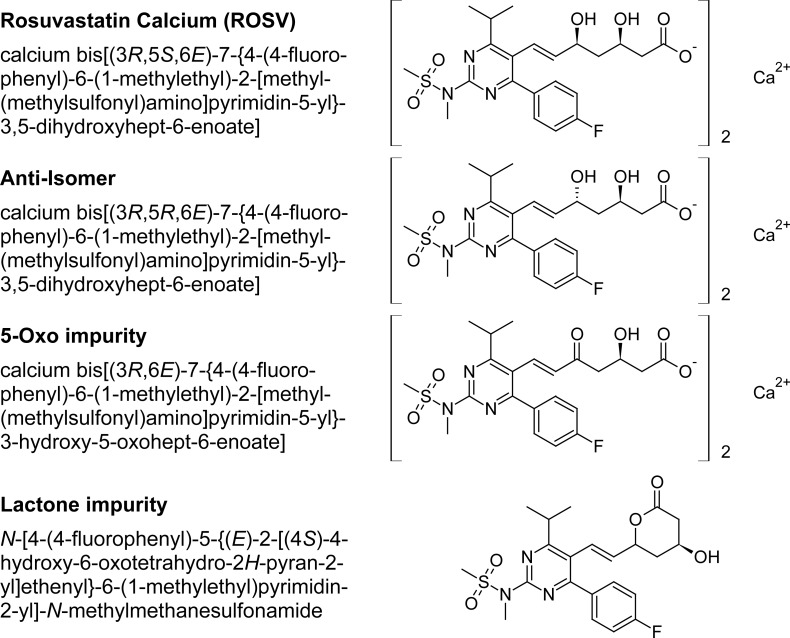
Chemical structures of ROSV, Anti-isomer, 5-Oxo impurity and Lactone impurity

Rosuvastatin (ROSV) is a synthetic lipid-lowering agent, chemically known as (3*R*,5*S*,6*E*)-7-{4-(4-fluorophenyl)-6-(1-methylethyl)-2-[methyl(methylsulfonyl)amino]pyrimidin-5-yl}-3,5-dihydroxyhept-6-enoic acid calcium salt (2:1). It is used for the treatment of hyperlipidemia and is an inhibitor of 3-hydroxy-3-methylglutaryl coenzyme (HMG-CoA) reductase. This enzyme catalyzes the conversion of HMG-CoA to mevalonate, an early and rate-limiting step in cholesterol biosynthesis [1]. ROSV calcium is a salt with pK_a_ of 4.6 and very slightly soluble in aqueous solutions of pH 4.0 and below. It exhibits a high degree of specificity for uptake into the liver and is a potent in vitro and in vivo competitive inhibitor of HMG-CoA reductase. The chemical structure of ROSV, Anti-isomer, Lactone and 5-Oxo are presented in Figure 1.

UPLC is a new category of separation technique based upon well-established principles of liquid chromatography which utilizes sub-2 μm particles for the stationary phase. These particles operate at elevated mobile phase linear velocities to affect dramatic increase in resolution, sensitivity and speed of analysis. Owing to its speed and sensitivity, this technique has gained considerable attention in recent years for pharmaceuticals and biomedical analysis. In the present work, this technology has been applied to the method development and method validation study of related substances determination of ROSV in pharmaceutical dosage forms.

**Fig. 2.**
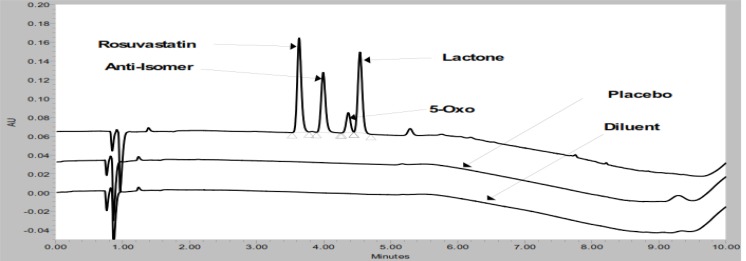
Overlaid chromatograms of placebo, diluent and standard (for identification)

A detailed literature survey for ROSV revealed that few analytical methods were reported for determination of ROSV by HP-TLC [2], UV spectroscopy [3–6], assay by RP-HPLC [7–11], capillary electrophoresis [12], Mass spectrometry [13–16], LC-MS/MS [17] and simultaneous determination with atorvastatin by mass spectrometry [18]. Mehta *et al.* reported a stability-indicating assay method for determination of ROSV in the presence of its degradation products using high performance liquid chromatography [19]. In this assay method total run time is around 35 min to elute all degradation impurities and is applicable for only ROSV estimation but not for its related substances. Gosulu VRR *et al.* reported a stability-indicating RP-UPLC method for ROSV and its related impurities in pharmaceutical dosage form [20]. In this method, total run time is 12 min to monitor all degradation products in ROSV dosage form. When forced degradation study (acid hydrolysis) of ROSV was performed using this reported method, three major late eluting impurities were observed after 12 min, which is presented in Figure 2. Currently, the determination of impurities is one of the most difficult tasks for pharmaceutical analysis during method development, especially if increasing numbers of impurities are required to be determined.

According to our knowledge, none of the currently available analytical methods can separate and quantify all the known related compounds, degradation impurities and unknown degradation compounds (late eluting) of ROSV dosage form in the claimed chromatographic run time. It’s indicated that published RP-HPLC and RP-UPLC methods are not suitable for the related substance determination in ROSV tablets dosage form, as per ICH guidance [21]. It is, therefore, necessary to develop a new stability-indicating method for the determination of ROSV related substances. Hence, we focused on developing a selective, fast, cost-effective, mass compatible and stability-indicating method using advance technique UPLC for the related substances determination of ROSV in solid pharmaceutical dosage form.

Hence, a reproducible, mass compatible stability-indicating RP-UPLC method was developed which is less time-consuming and more selective compared to the present methods, taking only 10 min for a single run. Developed method separates three known and six major unknown degradation products from each other and from ROSV within 10 min. Thereafter, the developed method was successfully validated according to International Conference on Harmonization (ICH) guidelines [21] to show the stability-indicating capability of the method.

## Results and Discussion

### Method development and optimization

The main criterion for developing an RP-UPLC method for the determination of related substances in ROSV dosage form in a single run, with emphasis on the method being accurate, reproducible, robust, stability-indicating, linear, free of interference from other formulation excipients and convenient enough for routine use in quality control laboratories.

**Fig. 3.**
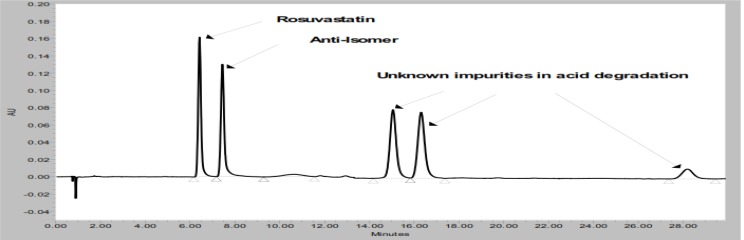
Acid degradation chromatogram of reference article

A spiked solution of impurities (5 μg/mL), ROSV (500 μg/mL) and placebo peaks were subjected to separation by RP-UPLC. Initially, the separation of all peaks was studied using 0.1% trifluoroacetic acid (TFA) as mobile phase A and methanol as mobile phase B on an Acquity BEH C18 (100 mm × 2.1 mm, 1.7 μm) column and Waters (UPLC) system with an isocratic program. The 0.3 mL/min flow rate was selected to achieve the separation of peaks. The column oven temperature was maintained at 40°C. These conditions resulted in separation of the ROSV peak with the placebo peaks and impurities peaks, represented in Figure 3. However, during the force degradation study some late elute peaks were observed. It is not incorporated with reference method. Based on obtained results, the isocratic program was replaced with the gradient program in an effort to achieve high resolution between the known impurities and all degradant peaks. With the Acquity UPLC C18 column (BEH (100 mm × 2.1 mm, 1.7 μm) column, different combinations of mobile phase A and B were studied to optimize the method, and the results of the optimization are summarized in Table 2, including any observations noted. From the mobile phase selection study, the optimized UPLC parameters were as follows: flow rate, 0.3 mL/min; column oven temperature, 40°C; injection volume, 7 μL; and a gradient program with mobile phase A and B. Based on the UV spectrum of the compound, 240 nm was found to be appropriate for the determination of ROSV impurities in pharmaceutical formulations. ROSV and all impurities are well resolved with respect to each other in a reasonable time of 10 min [Figure 2]. No chromatographic interference due to the blank (diluent) and other excipients (placebo) at the retention time of ROSV and all impurities were observed, as shown in Figure 2.

### Analytical parameters and validation

After development, this method was subjected to validation according to ICH guidelines. The method was validated to demonstrate that it is suitable for its intended purpose by the standard procedure to evaluate adequate validation characteristics (system suitability, accuracy, precision, linearity, robustness and stability-indicating capability).

### System suitability

The percentage relative standard deviation (RSD) of area from six replicate injections was below 5.0 % (diluted standard solution, 5μg/mL). Low values of RSD for replicate injections indicate that the system is precise. The results of other system suitability parameters such as peak tailing and theoretical plates are presented in Table 3. As seen from this data, the acceptable system suitability parameters would be as follows: the relative standard deviation of replicate injections is not more than 5.0 %, the tailing factor ROSV is not more than 1.5 and the theoretical plates are not less than 10000.

### Specificity

**Fig. 4.**
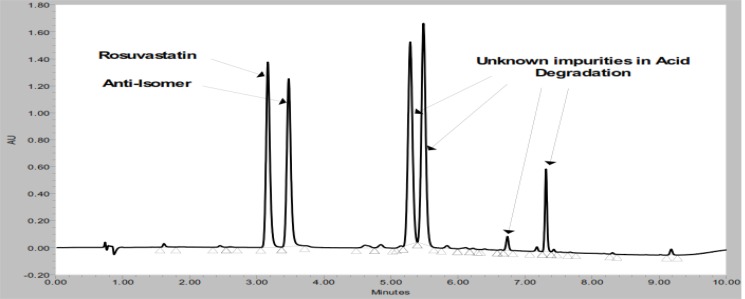
Acid degradation chromatogram of ROSV

Forced degradation studies were performed to demonstrate the selectivity and stability-indicating capability of the proposed RP-UPLC method. Figure 2 shows that there is no interference at the RT (retention time) of ROSV and all known impurities from the blank and other excipients. Significant degradation was not observed when ROSV was subjected to oxidation, base, thermal and hydrolytic, whereas significant degradation was observed when the ROSV was subjected to acid hydrolysis (0.1N HCl, 80°C, 2 h) and UV conditions, leading to the formation of rosuvastatin anti isomer and unknown impurities. The acid hydrolysis product (rosuvastatin anti isomer) and ROSV are well separated from each other, as seen in Figure 4. The peak attributed to ROSV was investigated for spectral purity in the chromatogram of all exposed samples and was found to be spectrally pure. The purity and related substances of ROSV were unaffected by the presence of other excipients and thus the stability-indicating power of this method is confirmed. The results of the forced degradation study are presented in Table 4.

### Limit of quantification (LOQ)

The concentration (in μg/mL) with a signal to noise ratio (S/N) of at least 10 was taken as the LOQ, which meets the criteria defined by ICH guidelines. The LOQ for the ROSV, rosuvastatin anti isomer, 5-oxo and lactone peaks was found to be 0.075 μg/mL. The precision was also established at the quantification level. The % RSD of the peak area was well within the acceptance limit of <10.0 %. The determined limit of qualification and precision at LOQ values for ROSV, rosuvastatin anti isomer, 5-Oxo and lactone are presented in Table 5.

### Linearity

**Fig. 5.**
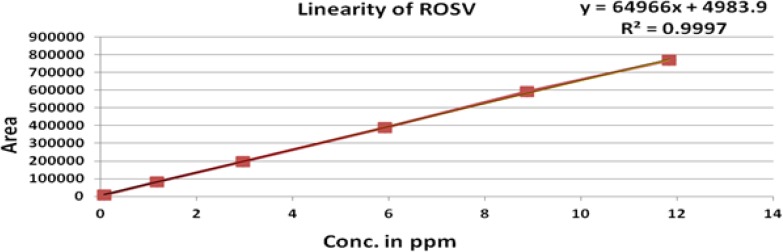
Linearity of Rosuvastatin

**Fig. 6.**
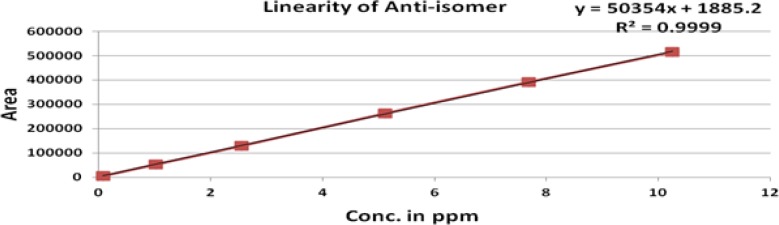
Linearity of Rosuvastatin Anti-isomer

**Fig. 7.**
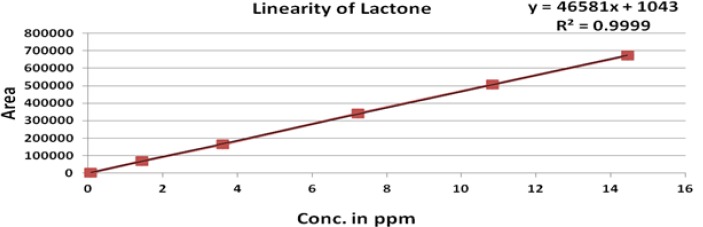
Linearity of Lactone

**Fig. 8.**
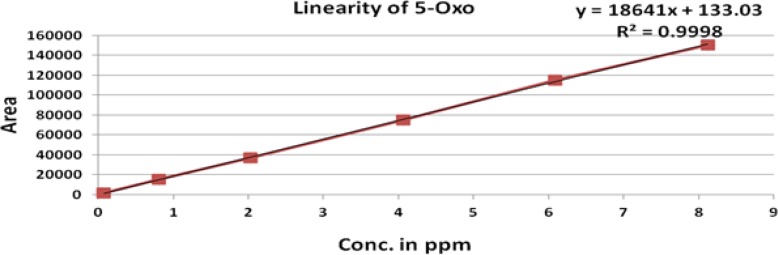
Linearity of 5-Oxo

The linearity of an analytical method is its ability to elicit test results that are directly, or by a well-defined mathematical transformation, proportional to the concentration of analyte in that sample within a given range. The response was found to be linear in the range of 0.075 to10 μg/mL. The regression statistics are shown in Table 6, with the linearity curve for ROSV, Anti isomer, 5-Oxo and Lactone represented in Figure 5–8.

### Precision

The purpose of this study was to demonstrate the reliability of the test results with variations. The results are shown in Table 7, along with intermediate precision data. Low RSD values indicate that this method is precise.

### Accuracy

The accuracy of an analytical method is the closeness of test results obtained by that method compared with the true values. The amount recovered (for LOQ, 50, 100 and 200 % level) was within ± 10 % of amount added; for the LOQ level, the amount recovered was within ± 20 % of the amount added, indicating that the method is accurate and that there is no interference due to other excipients present in the injection. The results of the recovery assay are shown in Table 8.

### Robustness

The robustness of an analytical procedure is a measure of its capacity to remain unaffected by small but deliberate variations in method parameters and provides an indication of its reliability during normal usage. No significant effect was observed on system suitability parameters such as RSD, tailing factor, or the theoretical plates of ROSV when small but deliberate changes were made to chromatographic conditions. The results are presented in Table 3, along with the system suitability parameters of normal conditions. Thus, the method was found to be robust with respect to variability in applied conditions.

## Experimental

### Materials and Reagents

Rosuvastatin tablets, impurities, rosuvastatin calcium API and placebo were provided by Cadila Pharmaceutical Ltd. Dholka, Ahmedabad, India, along with the working standard. Methanol (MeOH) of HPLC grade was received from J. T. Baker (NJ, USA). Trifluoroacetic acid (TFA) was obtained from Qualigens fine chemicals (Mumbai, India). All solutions were filtered through 0.2 μm nylon filters manufactured by Millipore Pvt. Ltd (Bangalore, India).

### Buffer preparation

A solution of 1% trifluoroacetic acid (TFA) was prepared using Milli-Q water. The buffer preparation was stable with respect to pH and maintained visual clarity for 48 h.

### Chromatographic conditions

Buffer (1 % TFA) was used as a mobile phase-A and methanol as a mobile phase-B in gradient program. System control, data collection and data processing were accomplished using Waters Empower chromatography data software. The analytical column used was 100 × 2.1 mm, 1.7 μm Waters Acquity UPLC BEH C-18 column (Milford, USA). The optimized conditions were as follows: injection volume: 7.0 μL, flow rate: 0.3 mL/min at a column temperature of 40 °C, sample cooler temperature: 10 °C and detection wavelength: 240 nm, gradient elution [Table 9]. Under these conditions the system back pressure was about 7200 psi. The stress degraded samples were analyzed using a PDA detector over a range of 200 – 400 nm.

### Diluent

Methanol was used as a diluent.

### Diluted standard solution preparation

The diluted standard solution was prepared by dissolving the rosuvastatin calcium standard and all three impurities in diluent to obtain a solution containing 5 μg/mL.

### Sample solution preparation

For the preparation, an equivalent of 50mg tablets of powder was accurately transferred into a 100 mL volumetric flask. Approximately 70 mL of diluent was added to the volumetric flask, which was then sonicated in an ultrasonic bath for 8 min. The resulting solution was then diluted up to the mark with diluent and mixed well.

### Placebo solution preparation

In preparing the placebo solution, 750 mg of placebo was accurately transferred into a 100 mL volumetric flask. Approximately 70 mL of diluent was added to the volumetric flask, which was then sonicated in an ultrasonic bath for 8 min. The resulting solution was then diluted up to the mark with diluent and mixed well.

### Method validation

The method described herein has been validated for related substances determination by UPLC.

### System suitability

System suitability parameters were performed to verify the system performance. System precision was determined on six replicate injections of standard preparations. All the important characteristics, including the relative standard deviation, peak tailing, and theoretical plate number, were measured.

### Specificity

Forced degradation studies were performed to demonstrate selectivity and stability-indicating capability of the proposed method. The sample was exposed to acidic (0.1 N HCl, 80°C, 2 h), alkaline (0.5 N NaOH, 80°C, 6 h), strong oxidizing (3 % H_2_O_2_, 80°C, 6 h), thermal (100°C, 8 h) and photolytic (UV) degradation conditions. All exposed samples and standards were then analyzed using the proposed method.

### Limit of quantification (LOQ)

The LOQ was determined using a signal to noise approach as defined in the International Conference on Harmonization (ICH) guidelines [21]. A serially diluted solution of rosuvastatin calcium and all impurities were injected into the chromatograph and the signal to noise (S/N) ratio were calculated at each concentration.

### Linearity

Linearity was demonstrated from 0.015% to 200 % of standard concentration using a minimum of six calibration levels (0.015 %, 50 %, 75 %, 100 %, 150 % and 200 %) for ROSV and all impurities. The method of linear regression was used for data evaluation. The peak areas of the standard compound were plotted against the respective standard concentration. Linearity was described by the linearity equation and the correlation coefficient was also determined.

### Precision

The precision of the system was determined using the 1 % spiked impurity sample preparation procedure described above for six real samples of rosuvastatin tablets and analysis using the same proposed method. Intermediate precision was studied using different columns and was performed on different days.

### Accuracy

To confirm the accuracy of the proposed method, recovery experiments were carried out by the impurities addition technique. Four levels (LOQ, 50 %, 100 % and 200 %) of standards were added to pre-analyzed samples in triplicate. The percentage recoveries of anti-isomer, lactone and 5-Oxo at each level and each replicate were determined. The mean of percentage recoveries (n = 9) and the relative standard deviation were also calculated.

### Robustness

The robustness is a measure of the capacity of a method to remain unaffected by small but deliberate changes in flow rate (± 0.03 mL/min), change in column oven temperature (± 5 °C) and change in wavelength nm (± 2 nm).

## Conclusion

A new RP-UPLC method was successfully developed for the estimation of related substances in rosuvastatin tablets. The method validation results have verified that the method is selective, precise, accurate, linear, robust and stability-indicating. The run time (10.0 min) enables rapid determination of impurities. This stability-indicating method can be applied for the determination of related substances in bulk drugs, pharmaceutical formulations and chemical processing. The developed method can also be applied for identification of unknown impurities.

## Figures and Tables

**Tab. 1.** Working concentration of Rosuvastatin and its impurities

**Compound**	**Working concentration**	
	**(mg/mL)**	**(μg/mL)**
ROSV	0.5	500
ROSV impurities	0.005	5

**Tab. 2.** Summary of method optimization

**Experimental condition**	**Observation**
Mixture of methanol and 0.1% TFA in the ratio of 50:50 employing isocratic elution; acquity UPLC BEH C18 column 100mm x 2.1 mm, 1.7μm; 40°C	During acid degradation study some late elute peaks were observed.
0.1% TFA (MP-A) and methanol (MP-B), linear gradient; acquity UPLC BEH C18 column 100mm x 2.1 mm, 1.7μm; 40°C	Satisfactory peak separation and peak shape observed within 10 min.

**Tab. 3.** System suitability results (precision, intermediate precision and robustness)

**Parameter**	Theoretical plates^*^	Tailing factor^*^	% RSD* of standard
Precision	13644	1.2	0.3
Intermediate Precision	13222	1.1	0.4
At 0.33 mL/min flow rate	15573	1.1	0.1
At 0.27 mL/min flow rate	12565	1.1	0.1
At 25°C column temp.	13980	1.1	0.2
At 35°C column temp.	13973	1.1	0.2
At 238 nm	13542	1.1	0.2
At 242 nm	12910	1.2	0.3

* …Determined on six values.

**Tab. 4.** Summary of forced degradation results

**Degradation condition**	**Peak Purity (ROSV)**	**Observation**
Control sample	Pass	Not applicable
Acid hydrolysis (0.1 N HCl, 80°C, 2 h)	Pass	Significant degradation
Alkaline hydrolysis (0.5 N NaOH, 80°C, 6 h)	Pass	No significant degradation
Oxidation (3 % H_2_O_2_, 80°C, 6 hours)	Pass	No significant degradation
Thermal (100 °C, 8 h)	Pass	No significant degradation
Exposed to UV	Pass	No significant degradation

**Tab. 5.** LOQ and its precision results

**Substance**	**LOQ (μg/mL)**	**S/N**	Precision (% RSD*)
ROSV	0.075	165.8	5.9
Anti isomer	0.075	77.0	6.1
5-Oxo	0.075	26.4	8.0
Lactone	0.075	60.7	7.7

* … Determined on six values

**Tab. 6.** Regression statistics

**Substance**	**Linearity range (μg/mL)**	**Correlation Coefficient (R^2^)**	**Y-intercept bias in %**
ROSV	0.075 to 11.84	0.999	1.4
Lactone	0.075 to 14.45	0.999	0.5
Anti isomer	0.075 to 11.25	0.999	0.9
5-Oxo	0.075 to 8.12	0.999	0.2

**Tab. 7.** Precision (5 μg/mL) and Intermediate precision (5 μg/mL) results for all impurities

**Substance**	**Precision**		**Intermediate precision**	

	**% impurity^#^**	% RSD*	**% Impurity^#^**	% RSD*
Anti-Isomer	1.074	0.5	0.967	2.3
Lactone	0.989	3.3	1.045	1.8
5-Oxo	1.087	0.4	1.021	2.6

# ... Average of six determinations;

* … Determined on six values

**Tab. 8.** Accuracy results

**Substance**		**At LOQ 0.075 μg/mL**	**At 50 % 2.5 μg/mL**	**At 100 % 5 μg/mL**	**At 200 % 10 μg/mL**
Anti-isomer	^#^Mean Accuracy	111.6	104.3	104.0	104.6
	%RSD*	6.6	0.4	0.4	0.2

5-Oxo	^#^Mean Accuracy	100.4	96.7	98.4	105.1
	%RSD*	11.5	1.0	0.3	0.4

Lactone	^#^Mean Accuracy	114.3	93.4	94.9	104.3
	%RSD*	10.1	0.5	1.4	0.3

* … Determined on three values;

# ... Mean of three determinations

**Tab. 9.** Gradient elution program

**Time in minutes**	**Mobile phase-A in %**	**Mobile phase-B in %**
0.0	45	55
3.5	40	60
6.5	15	85
7.5	15	85
7.6	45	55
10.0	45	55
